# Ischaemic stroke with multi-focal venous and arterial thrombosis due to hyperhomocysteinemia: anabolic androgenic steroid use and *MTHFR c.667 C > T* variant – a case report

**DOI:** 10.1186/s12883-023-03197-4

**Published:** 2023-04-26

**Authors:** JPK Chen, A Rees, CH Coughlan, W Goodison, E Murphy, A Chandratheva

**Affiliations:** 1 National Hospital for Neurology and Neurosurgery, London, UK; 2 University College London Hospitals NHS Foundation Trust, London, UK

**Keywords:** Case report, Ischaemic stroke, Venous thrombosis, Hyperhomocysteineaemia, Anabolic androgenic steroids

## Abstract

**Background:**

Severely elevated serum homocysteine is a rare cause of ischaemic stroke and extra-cranial arterial and venous thrombosis. Several factors can lead to mild elevation of homocysteine including dietary folate and B12 deficiency, and genetic variants of the methylenetetrahydrofolate reductase (MTHFR) enzyme. The use of Anabolic androgenic steroid (AAS) is under-reported, but increasingly linked to ischaemic stroke and can raise homocysteine levels.

**Case Report:**

We present a case of a man in his 40s with a large left middle cerebral artery (MCA) territory ischaemic stroke and combined multifocal, extracranial venous, and arterial thrombosis. His past medical history was significant for Crohn’s disease and covert use of AAS. A young stroke screen was negative except for a severely elevated total homocysteine concentration, folate and B12 deficiencies. Further tests revealed he was homozygous for the methylenetetrahydrofolate reductase enzyme thermolabile variant (*MTHFR c.667 C > T*). The etiology of this stroke was a hypercoagulable state induced by raised plasma homocysteine. Raised homocysteine in this case was likely multifactorial and related to chronic AAS use in combination with the homozygous *MTHFR c.677 C > T* thermolabile variant, folate deficiency and, vitamin B12 deficiency.

**Conclusion:**

In summary, hyperhomocysteinemia is an important potential cause of ischaemic stroke and may result from genetic, dietary, and social factors. Anabolic androgenic steroid use is an important risk factor for clinicians to consider, particularly in cases of young stroke with elevated serum homocysteine. Testing for *MFTHR* variants in stroke patients with raised homocysteine may be useful to guide secondary stroke prevention through adequate vitamin supplementation. Further studies looking into primary and secondary stroke prevention in the high-risk MTHFR variant cohort are necessary.

## Background

Severely elevated serum homocysteine is a rare cause of ischaemic stroke and extra-cranial arterial and venous thrombosis. Several factors can lead to mild elevation of homocysteine including dietary folate and B12 deficiency, and genetic variants of the methylenetetrahydrofolate reductase (MTHFR) enzyme. Use of Anabolic androgenic steroid (AAS) is under reported, but increasingly linked to ischaemic stroke and can raise homocysteine levels.

## Case report

**Fig. 1:**
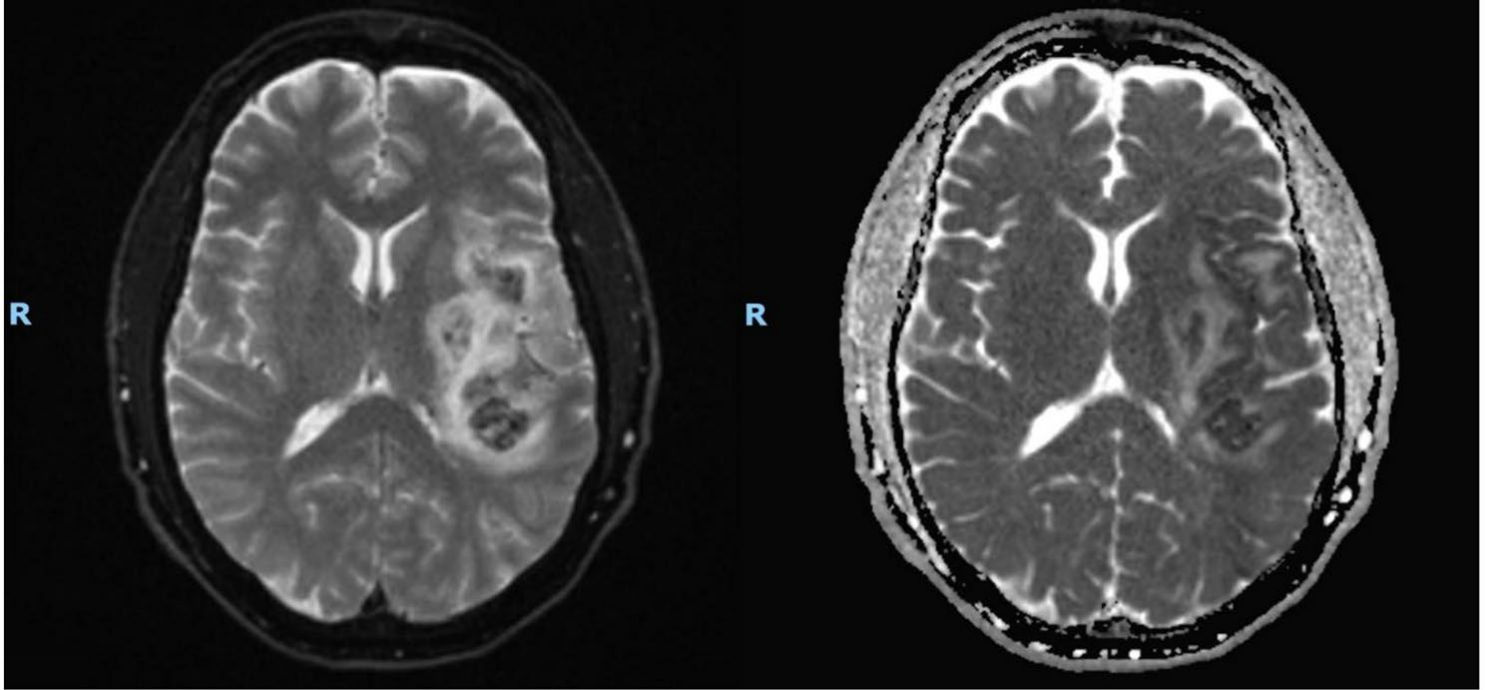
MRI imaging performed 9 days following left MCA territory stroke. Diffusion-weighted imaging (DWI) left and corresponding apparent diffusion coefficient (ADC) right

We present a case of a 49-year-old male repatriated back to the United Kingdom (UK) following a left middle cerebral artery (MCA) territory ischaemic stroke which occurred whilst abroad. The initial National Institute of Health Stroke Scale (NIHSS) was 15. His primary deficits were a severe mixed aphasia and verbal apraxia (NIHHS 2), with difficulty following two-step commands and impaired comprehension at the sentence level. He had a partial right facial paralysis (NIHSS 2) with severe right arm and leg weakness (NIHSS 4 and 2 respectively). Left MCA infarct involving frontal, temporal, and parietal lobes was demonstrated on initial CT and MRI imaging (Fig. 1). He was also found to have both a pulmonary embolism on CT pulmonary angiography and a right popliteal artery thrombus on lower limb ultrasonography.

He had a diagnosis of Crohn’s disease, that was well controlled with azathioprine and for which a colectomy and ileostomy were performed over ten years prior. There was no evidence of active Crohn’s colitis during his admission. He did not smoke or drink alcohol. However, after his stroke, it came to light that he had engaged in covert persistent anabolic androgenic steroid (AAS) use over six years for body building.

An extensive young stroke screen based on local stroke unit guidelines was completed including normal lipid and Hba1c profiles, negative thrombophilia panel including anti-phospholipid antibodies, negative HIV, hepatitis, syphilis, and autoimmune vasculitis screens. *JAK2* gene testing, alpha-galactosidase activity (Fabry disease), and paroxysmal nocturnal hemoglobinuria screen were also normal.

Carotid doppler studies, interval MRI and CT did not reveal any intracranial vascular abnormalities. Investigations for cardioembolic stroke including trans-thoracic and bubble echocardiography, and prolonged cardiac monitoring were negative. A CT-PET was performed that was negative for malignancy and Crohn’s disease activity.

He had an elevated total homocysteine concentration of 87 µmol/L (Reference range (RR): 5–12 µmol/L) following the acute infarct with a corresponding normal methylmalonic acid 0.11µmol/L (RR: <0.29 µmol/L), low vitamin B12 74 pg/mL (RR: 160–800 pg/mL), and low folic acid level < 2.2ng/mL (RR: >2.7 ng/mL). Serum testosterone was suppressed at 0.8nmol/L (RR: 10-30nmol/L) secondary to presumed exogenous steroid use. Subsequent genetic testing for methylenetetrahydrofolate reductase (*MTHFR*) variants showed that he was homozygous for the common *c.677 C > T MTHFR* thermolabile variant.

His admission was complicated by significant agitation due to a combination of AAS withdrawal, and combined aphasia. He was temporarily commenced on risperidone (1 mg daily). His initial antiplatelet medication was converted to apixaban due to his pulmonary and peripheral artery emboli. He was treated with hydroxocobalamin (1 mg intramuscular 3 monthly) and folate replacement (5 mg once daily), both of which normalized along with homocysteine.

**Fig. 2:**
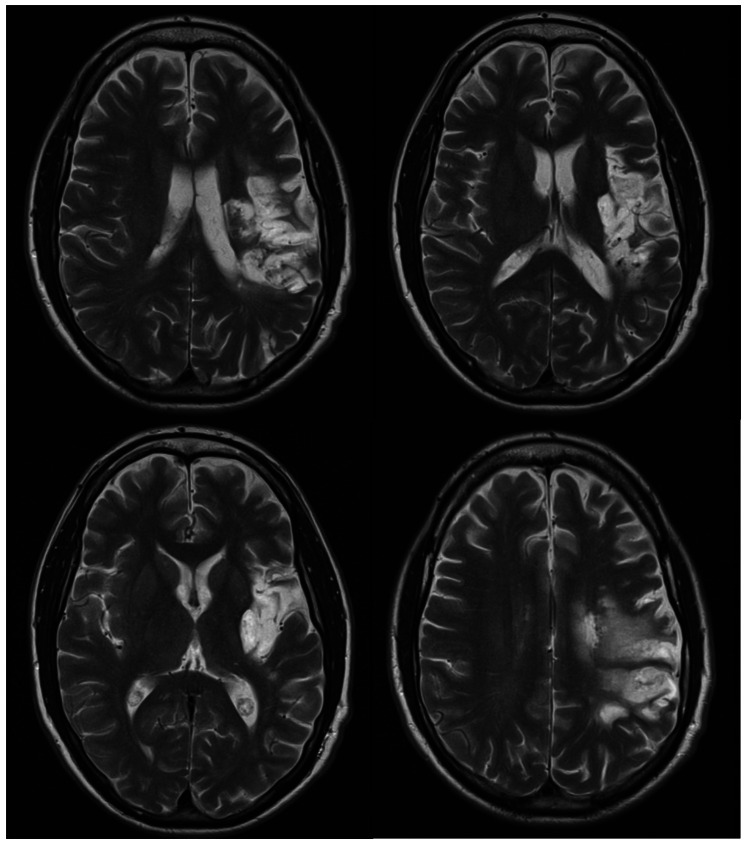
Interval Axial T2 MRI scan performed 5 months following acute stroke. Indicating mature large left MCA territory infarct

He made significant improvements following several months of stroke rehabilitation. Interval MRI imaging demonstrated a mature left MCA infarct (Fig. 2). Prior to discharge he was self-caring and mobilizing independently. He had persistent speech apraxia and limited verbal output but was able to communicate using written methods. He was repatriated to a local hospital awaiting social care input and ongoing speech and language therapy.

## Discussion

The etiology of this large ischaemic stroke and combined venous and arterial thrombosis is likely due to a hypercoagulable state, induced by significantly elevated levels of total homocysteine. In this patient, this is likely multifactorial and related to chronic anabolic steroid use in combination with the homozygous *MTHFR c.677 C > T* thermolabile variant, folate deficiency, and vitamin B12 deficiency.

Folate and vitamin B12 deficiency was attributed to dietary insufficiency and possibly contributed by a history of Crohn’s disease. However, in this case, there were no clinical or CT-PET findings to suggest active disease. Cessation of AAS in combination with vitamin B12 and folate replacement led to normalization of homocysteine concentration on repeat testing.

## Homocysteine & stroke

Homocysteine is a toxic intermediate of the metabolism of the amino acid methionine, low levels are therefore typically maintained in plasma[1]. Ineffective intracellular metabolism of homocysteine leads to an increase in its plasma concentration. Homocysteine metabolism is dependent on sufficient Vitamin B6, B12, folate, and the function of the MTHFR enzyme. The recycling of homocysteine to methionine is dependent on both the MTHFR enzyme and folate as essential co-factors[2]. Hyperhomocysteinemia is one of the few conditions that may present with both arterial and venous thrombosis. The dose-dependent relationship between homocysteine concentration and risk of ischaemic stroke, as well as extracranial arterial and venous thromboembolism, is well characterized [3, 4]. Homocysteine has been shown to induce endothelial dysfunction, promote oxidative stress, and induce coagulation abnormalities such as reduced activity of tissue plasminogen activator (tPA) and increased activation of Plasminogen activator inhibitor-1( PAI-1) [5–7]. Chronic mild elevations of homocysteine may also promote large and small vessel atherosclerosis leading to ischaemic stroke [7].

## Anabolic steroids & stroke

AAS use is common in the UK and is likely under-reported; in our case this was covert and only subsequently revealed. Anabolic androgenic steroid use leading to ischaemic stroke has been previously reported but primarily limited to case reports [8–10]. Proposed prothrombotic mechanisms include dyslipidemia, platelet aggregation, and increased hematopoiesis resulting in erythrocytosis [11–13]. A study by Graham et al. demonstrated chronic anabolic steroid use can raise homocysteine levels compared to controls independent of B12 and folate concentration[14]. Elevation of homocysteine by AAS may contribute to stroke risk, however, the exact mechanism by which AAS leads to elevated homocysteine is unknown [12, 14].

## MTHFR & stroke

Several known variants of the *MTHFR* gene exist leading to reduced activity of the enzyme MTHFR. A thermolabile variant was first discovered in 1988 caused by a missense mutation, *c.677 C > T (rs1801133)* [15]. This variant is common with significant differences between ethnic groups, with the highest prevalence in White and Hispanic populations [16]. The estimated global prevalence of heterozygotes is between 30 and 50% and homozygotes between 3 and 15% (16). Homozygotes with this *MTHFR* variant have an enzymatic function at roughly 30% compared to those without the variant [17]. Homozygosity is associated with a mild elevation of homocysteine and increased stroke risk compared to heterozygotes and controls. The extent in which *MTHFR* variants contribute to stroke risk has been inconsistent or thought to be moderate compared to the classical stroke risk factors[18].

## Lowering homocysteine & stroke prevention

Plasma homocysteine can be reduced through treatment with folic acid supplementation. However, the results of large trials for stroke prevention with B vitamins and folic acid supplementation are inconsistent and largely negative [19–21]. Analysis of stroke outcomes from the Heart Outcomes Prevention Evaluation 2 trial did suggest a reduction in stroke incidence with folic acid supplementation in sub-groups without routine folic acid fortification in food and sub-groups with higher baseline homocysteine levels [20]. Furthermore, a meta-analysis of folic acid supplementation in countries without routine fortification in foods was associated with a 25% risk reduction in stroke [22].

It is suggested that patients with *MTHFR c.677 C > T variants* may have increased folate requirements to maintain normal serum folate and homocysteine concentrations[23]. The China stroke primary prevention randomized control trial demonstrated reduced stroke risk with folic acid replacement in the *MTHFR* variant population [24]. Therefore the *MTHFR* variant sub-group may represent a high-risk group for intervention [23]. Routine testing of homocysteine which is typically reserved for young stroke presentations in the UK may be a useful screen to guide further *MTHFR* gene testing as part of our stroke management.

## Conclusion

In summary, hyperhomocysteinemia is an important potential cause of ischaemic stroke and may result from genetic, dietary, and social factors. Anabolic steroid use is an important risk factor for clinicians to consider, particularly in cases of young stroke with elevated serum homocysteine. Testing for *MFTHR* variants in stroke patients with raised homocysteine may be useful to guide secondary stroke prevention through adequate vitamin supplementation. Further studies looking into primary and secondary stroke prevention in the high-risk MTHFR variant cohort are necessary.

## Data Availability

N/A.

## Abbreviations

MTHFR: Methylenetetrahydrofolate reductase
AAS: Anabolic androgenic steroid
MCA: Middle cerebral Artery
UK: United Kingdom
NIHSS: National Institutes of Health Stroke Scale
